# Underwater 3D Surface Measurement Using Fringe Projection Based Scanning Devices

**DOI:** 10.3390/s16010013

**Published:** 2015-12-23

**Authors:** Christian Bräuer-Burchardt, Matthias Heinze, Ingo Schmidt, Peter Kühmstedt, Gunther Notni

**Affiliations:** 1 Fraunhofer Institute Applied Optics and Precision Engineering, Albert-Einstein-Str. 7, D-07745 Jena, Germany; matthias.heinze@iof.fraunhofer.de (M.H.); ingo.schmidt@iof.fraunhofer.de (I.S.); peter.kuehmstedt@iof.fraunhofer.de (P.K.); gunther.notni@iof.fraunhofer.de (G.N.); 2 Technical University Ilmenau, Ehrenbergstraße 29, D-98693 Ilmenau, Germany

**Keywords:** underwater 3D-scanner, fringe projection, underwater stereo camera calibration

## Abstract

In this work we show the principle of optical 3D surface measurements based on the fringe projection technique for underwater applications. The challenges of underwater use of this technique are shown and discussed in comparison with the classical application. We describe an extended camera model which takes refraction effects into account as well as a proposal of an effective, low-effort calibration procedure for underwater optical stereo scanners. This calibration technique combines a classical air calibration based on the pinhole model with ray-based modeling and requires only a few underwater recordings of an object of known length and a planar surface. We demonstrate a new underwater 3D scanning device based on the fringe projection technique. It has a weight of about 10 kg and the maximal water depth for application of the scanner is 40 m. It covers an underwater measurement volume of 250 mm × 200 mm × 120 mm. The surface of the measurement objects is captured with a lateral resolution of 150 μm in a third of a second. Calibration evaluation results are presented and examples of first underwater measurements are given.

## 1. Introduction

Recently, the acquisition of 3D surface geometry of underwater objects has attracted increasing interest in various research fields, including archaeology [1,2,3,4], biological applications [5,6,7,8], or industrial facility inspection tasks [9]. Typical 3D reconstruction methodologies for underwater applications are underwater photogrammetry [5,7,10,11,12,13] or laser scanning techniques [9,14,15,16,17]. These techniques are time consuming and have limited precision. The increasing requirements of modern application scenarios concerning accuracy and data acquisition speed, however, require new approaches. One such approach is the use of structured light for the illumination of the underwater scene and to capture 3D measurement data using a stereo camera rig. Structured light illumination should be conducted by projection of a certain pattern (for example fringe pattern, stochastic pattern, other patterns) onto the object to be measured.

Recently, structured light (fringe) projection-based techniques have been increasingly used for underwater 3D measurements [18,19,20]. Some advantages of this technique in contrast to classical photogrammetric measurements are obvious: more measurement points can be generated and a higher 3D point density can be obtained in the measurement result. Additionally, measurement data can be obtained quickly and automatically. Due to the imaging principle, improved measurement accuracy can be expected. The main disadvantage, however, is the restricted measurement field because of the limited illumination conditions under water. Although the small size of the measurement field is a real limitation for certain potential applications we propose a fringe projection based method for underwater 3D data acquisition. Additionally, we introduce a prototype of a handheld underwater 3D scanning device in order to show the power of the structured light projection technique for underwater 3D measurements.

The main motivation for our developments is high precision inspection of industrial facility parts located under water (see e.g., [21]) such as pipeline systems, tanks, and pylons of off-shore windmills. Here a structured light based inspection methodology can help to carry out the maintenance of such facilities with considerably lower effort.

However, underwater 3D measurements using the structured light projection technique are more complicated than measurements in air. Reasons are the light absorbing property of the water, possible water pollution, and light ray fraction at the interfaces of different media (water, glass, air). Other causes are the more sophisticated requirements of the mechanics concerning robustness against impacts, temperature, humidity, and other environmental influences.

Our first goal was the construction of a new, handheld, practicable 3D scanning device for underwater measurements including algorithms for the attainment of accurate measurement results, and the development of a calibration procedure which can be done with low effort, but makes accurate measurement results possible. The second goal was to show the suitability of the structured light projection technique for sophisticated measurement tasks in underwater environments.

In this paper, an overview over the state of the art in optical underwater 3D measurement techniques is given, the challenges of the application of the structured light projection technique are outlined, the principles of accurate underwater 3D measurements are introduced, the development of a new handheld scanning device is described, and the first experimental results are presented and discussed.

## 2. State of the Art

Photogrammetry has been applied to underwater measurements with increasing importance and frequency for more than 40 years [22,23]. Documentation of archaeological sites [1,2] or sunken objects like boat parts or ship wrecks [10] are some application fields, as well as inspection and surface measurement of industrial facilities (for example pipeline systems [9]) or the measurement of biological objects such as coral reefs [8] or fishes [5,6,7].

The challenges for photogrammetric underwater applications are mainly the robustness and the accuracy of the captured 3D data. This is due to potentially bad image recording conditions (polluted water, insufficient light sources, difference in absorption behavior of distinct wavelengths, *etc.*) on the one hand and the possibly limited quality of the photogrammetric sensor calibration on the other hand. Water quality and illumination conditions have considerable influence on the measurement result and have been examined by Sedlazek *et al.* [24], Schechner and Karpel [25], and Bianco *et al.* [20].

Many works are concerned with calibration of photogrammetric underwater stereo scanners. An overview is given by Sedlazeck and Koch [12]. There are works considering the significant refraction effects using plane interface glasses of the underwater sensor housing [11,13,26,27,28,29]. Other authors neglect the refraction effects and try to approximate the underwater situation using the common pinhole camera model [5,7,30,31,32]. They propose modifications concerning the principal distance and the lens distortion [33,34].

A third situation appears when spherical dome ports are used in the underwater housing and the projection centers of the cameras are placed exactly into the sphere center points. Then, theoretically, no refraction occurs because of the perpendicular crossing of the interfaces of all vision rays. In this case, the common pinhole model may be applied for underwater applications in the same way as in the normal case. Furthermore, the calibration procedure can be performed under “normal” laboratory conditions in air. However, if the necessary preconditions concerning the exact spherical form of the dome-ports, the homogeneity of the glass material, and the exact placement of the cameras are denied, then measurement errors occur which should be corrected by a suitable method, as for instance additional distortion correction. Authors who have used this model describe a considerable deviation of the expected parameters [20].

The decision concerning the camera model used should depend on the requirements of the measurement accuracy and the accepted calibration effort. Here application of a suitable evaluation strategy seems meaningful which could be the determination of the measurement error using a certain reference measurement object with known geometry.

The use of structured light for photogrammetric underwater measurements has been recently proposed by Bruno *et al.* [18], Zhang *et al.* [19], and Bianco *et al.* [20], respectively. They showed that this technique can be successfully applied to underwater measurements. However, there are still some restrictions (small measurement fields and short measurement distances) of the structured light technique which should be overcome in the near future.

The present work describes the application of the structured light technique using projection of fringe sequences (Gray-code and sinusoidal sequences) in two orthogonal projection directions and the development of the prototype of an underwater measurement device which could be useful for certain applications and should be developed further for more sophisticated applications.

## 3. Measurement Principles and Underwater Challenges

### 3.1. Basic Principles

Contactless optical 3D measurement using active or passive photogrammetric methods employing a stereo camera arrangement will be performed as follows: the measurement object will be recorded by two cameras with known intrinsic parameters (principal point, principal distance, and distortion function) and known relative orientation to each other (extrinsic parameters). The intrinsic and extrinsic camera parameters are obtained by calibration according to the selected camera model (see next paragraph).

Measured 3D points are computed by triangulation (see e.g., [35]) which means the intersection of two rays in the 3D space. If the two rays are not parallel, there exists one unique 3D point which is either a proper intersection point of the two rays or that point having the shortest Euclidean distance to both rays. The vision rays can be obtained by the image co-ordinate and the intrinsic and extrinsic camera parameters. In common air applications typically the well-known pinhole camera model (PM) will be used [35]. However, also other, more general camera models can be applied such as the “raxel” model [36] or the ray-based camera model (RM) as described by Bothe *et al.* [37]. Similarly, whether the ray-based model is used or the pinhole model, calculation of resulting 3D points is obtained by triangulation.

### 3.2. Correspondence Finding and 3D Point Calculation

The determination of point correspondences may be achieved with the help of markers on the measurement object or by finding some characteristic structure(s) in the images. By using structured illumination one projects such characteristic structures onto the measurement object and increases significantly the number of correctly identifiable corresponding points. It additionally provides the possibility of better sub-pixel exact determination of image co-ordinates of the corresponding points. The projected patterns may be sequences of Gray-codes, sinusoidal fringe patterns, or series of stochastic patterns (see e.g., [38,39,40,41,42]).

The principle of fringe projection based sensors for the 3D surface acquisition is called phasogrammetry [39] or fringe projection profilometry [40] and means the mathematical conjunction of fringe projection and photogrammetry. A projection unit produces sequences of fringe pattern which will be observed by one or more cameras and transformed into so called phase images. Using stereo camera equipment as in our case, the phase images may serve purely for the identification of corresponding points.

From the sequence of sinusoidal fringe images the rough phase will be calculated which is periodically distributed according to the period length over the image. The Gray-code enables the unique identification of the period number and leads together with the rough phase to the fine or unwrapped phase value. Determination of the phase values in two orthogonal projection directions [39] or the use of epipolar geometry information [35] make the phase values unique. Because the epipolar geometry can be applied only approximatively in case of refracted rays in underwater application, two perpendicular sequences of fringes should be used. In the case of underwater application the triangulation is performed between the vision rays in the water together with the calibration parameters of a virtual camera (see Section 3.3).

### 3.3. Extended Camera Model

**Figure 1:**
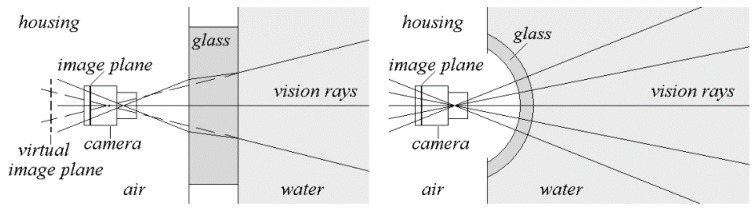
Geometry of rays in case of plane glasses (**left**) and spherical dome ports (**right**).

In contrast to classical photogrammetric modeling the pinhole camera model is not valid for underwater use because of the fact that there is no single viewpoint (the projection center) where all the image points representing rays pass through. This happens because of the refraction of the vision rays at the interfaces between air and housing glass (viewport) inside and between viewport and water outside the scanner housing (see Figure 1) following Snell’s law. An exception would be the usage of spherical dome ports and an exact positioning of the camera’s projection center into the center-point of the sphere (see Figure 1). Here (theoretically) no refraction occurs, and the pinhole model can be used as well as the same camera parameters as in air. However, in practice the exact positioning of the cameras is difficult to achieve. Experiments of other researchers [10,20] have showed considerable differences in the calibration parameters when performed both in air and under water. These differences lead to additional errors and distortion effects which must be corrected correspondingly.

**Figure 2:**
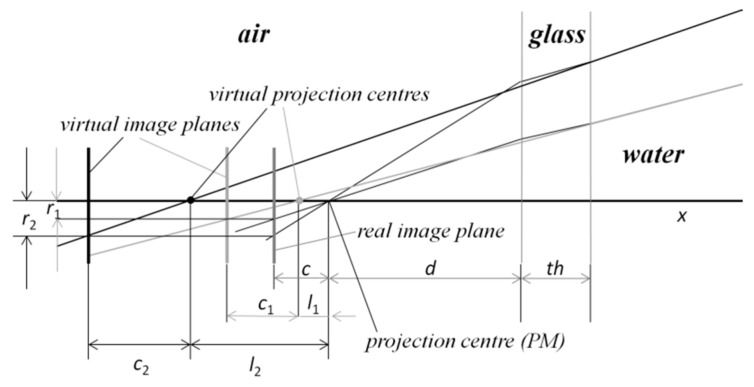
Ray geometry for two image points with radial distances *r*_1_ and *r*_2_ from the principal point using plane glasses (according to [44]).

Let us consider the case of plane interface ports again. The refraction at the two interfaces leads to the effect that the vision rays corresponding to the different image points do not intersect in one single point (the projection center according to PM). Additionally, the principal distance *c* becomes image point dependent. For illustration see Figure 2. The refraction of the rays is shown for the case of perpendicular orientation of the camera according to the glass interface.

In this case the pinhole camera model can be applied only as an erroneous approximation. In order to avoid these errors the refraction should be taken into account. The description of the camera model can be given accordingly to the ray-based model given by Bothe *et al.* [37] or corresponding to other proposals (see e.g., [11,13,26,28,33,43]).

Our approach is to use the parameters of the air calibration and describe the direction of the vision rays according to refraction and the additional parameters glass thickness *th* and interface distance *d*. Hence, one task of the underwater calibration is to find the values for the additional parameters. The other one is the formulation of the correct calculation of 3D measurement points based on found corresponding image points in the two cameras.

### 3.4. Approach for Underwater 3D Measurement

Our new approach to obtain accurate underwater 3D measurements using structured light illumination is the following. The exact modeling of the geometric situation of the cameras should be performed by consideration of the refraction effects and use of an accurate air calibration of the stereo scanner. An additional requirement is a relative low effort of the calibration procedure. This means that only few recordings of certain calibration objects should be acquired under water as input of the calibration procedure. The calibration for the underwater stereo scanner will be performed by the following steps:

1. Air calibration using the pinhole model without underwater housing
2. Determination of additional parameters (refraction indices, glass thickness, interface distance)
3. Ray-based model description of the underwater scanner inside housing
4. Determination of additional distortion matrices

When calibration is complete, 3D measurements can be performed under water. The 3D surface data of the measurement objects are obtained by finding corresponding points in the two camera images and performing a triangulation using the extended camera model and the parameters obtained by calibration. In the next section we will describe the calibration process in detail.

### 3.5. Calibration Procedure

The calibration of the underwater scanner can be performed according to the steps described in the previous section. For the first step any suitable calibration procedure for stereo camera scanners using the PM including distortion description can be used. For the second step we suggest the following procedure: our approach is to use the parameters of the air calibration and describe the direction of the vision rays according to refraction and the additional parameters interface distance *d* and glass thickness *th*. The indices of refraction for air *n_a_*, water *n_w_*, and glass (acrylic) *n_g_* are assumed to be sufficiently exact known. The camera orientations concerning the glass interfaces are supposed to be perpendicular. Possible deviations should be compensated by final application of the additional distortion matrix.

Hence, one task of the underwater calibration is to find the values for the additional parameters. The other one is the formulation of the correct calculation of 3D measurement points based on found corresponding image points in the two cameras.

We assume for simplification an orthogonal normal angle regarding the glass surface. By consideration of the geometry as depicted in Figure 2 we get a shift *l* of the projection center in direction of the optical axis and a modified principal distance *c*’ depending on the Euclidean distance *r* of the image points to the principal point:

(1) $$l\left(r\right)=\frac{d\cdot r}{c\cdot \text{tan}\left(\text{arcsin}\left(\frac{\text{sin}\left(\text{arctan}\left(r/c\right)\right)}{n_{w}}\right)\right)}-d-th\cdot \frac{r}{c}\cdot \left(\frac{r}{c}-\text{tan}\left(\text{arcsin}\left(\frac{\text{sin}\left(\text{arctan}\left(r/c\right)\right)}{n_{g}}\right)\right)\right)$$

(2) $$c\prime \left(r\right)=\frac{r}{\text{tan}\left(\text{arcsin}\left(\frac{\text{sin}\left(\text{arctan}\left(r/c\right)\right)}{n_{w}}\right)\right)}$$

Equations (1) and (2) are applied at calculation of the 3D points by triangulation complementing the known formulas of the pinhole camera case. By this extension we, in fact, obtain a ray-based camera model (RM).

With Equations (1) and (2) we can uniquely describe the rays corresponding to the image points *q* = (*x*, *y*) according to the RM by the two 3D points *O_i_*(*x*, *y*) and *Q_i_*(*x*, *y*).

(3) $$O_{i}\left(x,y\right)=O_{i}-l\left(r\right)\cdot R\cdot e$$

(4) $$Q_{i}\left(x,y\right)=O_{i}\left(x,y\right)-c\prime \left(r\right)\cdot R\cdot \left(\begin{array}{c} x\\ y\\ -c\prime \left(r\right) \end{array}\right)$$

where *O_i_* is the initially determined projection center of the camera outside housing according to the pinhole model, e is the unit vector, and R is the rotation matrix of the air calibration (see also [45]). For simplification we assume without loss of generality no distortion here.

The next step is the determination of the glass thickness *th*. In our case we measured it tactile before mounting the underwater housing. If this would not be possible, e.g., the method proposed by Chen and Yang [46] can be applied.

**Figure 3:**
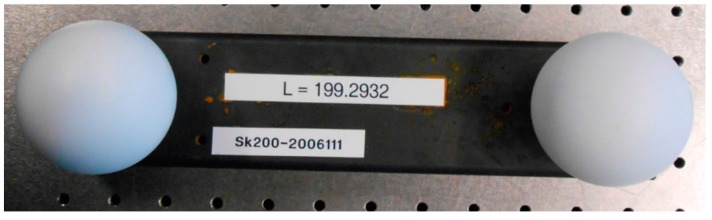
Specimen for calibration: calibrated ball bar.

Finally, the interface distances *d*_1_ and *d*_2_ for both cameras had to be determined. In order to obtain this, the following algorithm for underwater interface distance determination (UIDD) using four measurements (*M*_1_, *M*_2_, *M*_3_, and *M*_4_) is applied:

- Underwater recording (*M*_1_, *M*_2_) of specimen with known (calibrated) length information *L_r_* (ball bar—see Figure 3) in two different positions
- Underwater recording (*M*_3_, *M*_4_) of a plane surface in two different distances (minimum and maximum distance in the measurement volume) according to the scanner
- Definition of the error function for the test statistics *T* utilizing length measurement error and flatness error of the plane according to Equations (6) and (7)
- Determination of the searched parameters by minimization of *T*

Minimization of *T* can be achieved by a systematic search in the parameter space of *d*_1_ and *d*_2_ with meaningful search interval limits and step-widths. Having only two parameters, systematic search may be considerable more effective than trying to formulate an optimization function, because of the trigonometric functions in the Equations (1) and (2). The test quantity *T* is defined as:

(5) $$T\left(d_{1},d_{2}\right)={\left(E_{L,M1}\right)}^{2}+{\left(E_{L,M2}\right)}^{2}+{\left(E_{F,M3}\right)}^{2}+{\left(E_{F,M4}\right)}^{2}$$

with relative length error *E_L_* and relative flatness error *E_F_* defined as:

(6) $$E_{L}=\left|\frac{L_{a}-L_{r}}{L_{r}}\right|$$

and:

(7) $$E_{F}=\frac{\left|d_{\text{max}}\right|+\left|d_{\text{min}}\right|}{d_{r}}$$

Here, *L_r_* is the calibrated reference distance between the two spheres of the ball bar and *L_a_* is the measured distance. Quantity *d_r_* represents the maximal possible distance in the observed region (e.g., diameter of a circle area or diagonal of a rectangular region) at the plane measurement. It is a constant which is fixed according to the measurement field size and which should have a length of at least half the diagonal of the measurement volume. The term |*d_max_*| + |*d_min_*| denotes the flatness measurement error, which is defined as the range of the signed distances of the measured points from the best fit plane as defined in [47]. Application of this algorithm leads to the searched parameters *d*_1_ and *d*_2_. A more detailed description of the calibration procedure can be found in [44]. For all measurements it is allowed to remove a maximum of 0.3% outliers according to [46].

Application of this calibration procedure yielded good and meaningful results (see Section 5). However, the *E_F_* error seemed to be too high, and the shape deviation of the plane was quite systematic (bowl-shape). This led to the introduction of an additional or alternative, respectively, distortion function and a corresponding matrix operator *D* for distortion correction. This operator will be obtained as follows. The two plane measurements (*M*_3_ and *M*_4_) are used for the determination of *D*. For both a fitting of a plane to the 3D points is performed leading to the plane parameters E_1_ = {A_1_, B_1_, C_1_, D_1_} and E_2_ = {A_2_, B_2_, C_2_, D_2_}, respectively. Now, every calculated 3D point *P* is replaced by the nearest point *P*’ in the fitted plane. Using *P*’, new residuals for the corresponding image points are determined. Finally, the new distortion function is obtained by approximation of the residuals (from both planes) by a polynomial function of third degree leading to ten parameters (see [47] for more details).

In order to compensate new arising scaling errors after application of the new distortion compensation operator, last step of UIDD algorithm is performed again. If necessary, this procedure can be performed iteratively. The improvement in the resulting calibration is described in Section 5. Additionally, first results of the underwater measurements are presented.

## 4. New Underwater 3D Scanner

The paragon for the inner parts of the scanner was the handheld “kolibri Cordless” mobile scanning device [48]. For the underwater application, however, certain functional units such as the PC technique, the included display, and considerably more robust mechanics had to be developed completely anew. Additionally, an underwater resistant housing was necessary. The desired technical system parameters were similar to those of the “kolibri Cordless” and are listed in Table 1.

**Table 1:** Desired system parameters.

Property	Desired Parameter
Measurement volume (MV)	250 × 200 × 150 mm^3^
Working distance	500 mm
Camera resolution	1600 × 1200 pixel
Lateral resolution in the MV	150 μm
Noise of the 3D points	10 μm … 50 μm
Frame rate	60 Hz
Recording time per scan	350 ms
Maximal water depth	40 m
Sensor weight (without housing)	2 kg
Sensor weight with housing	10 kg

A new principle is the direct connection and insertion of the control and analysis PC and the display into the underwater housing. This allows the omission of a connection between the scanner and an external PC, which would be otherwise carried in a backpack or external case. Additionally, underwater handling of the scanner becomes easier. The consequently higher weight of the device is no disadvantage in underwater use because it is only important that neither sinking nor upwelling of the scanner occur. The following criteria were essential for the scanner construction:

- Desired technical system parameters (see Table 1)
- Easy handling at use and also at the process of mounting into the housing
- Compactness of the scanner including housing
- Low weight
- Suitable heat exhausting at underwater use
- Easy navigation of the scanner under water

The main components of the scanner, which should be connected compactly for mounting into the housing, are the projection unit, two cameras, the PC, the display, cooling elements, and mechanical elements. The projection unit including the lens causes the structured illumination of the scene which is observed by the two cameras. The PC controls the projection and observation, stores the recorded image data, and performs data processing. A rough presentation of the resulting 3D point cloud on the display allows the user to evaluate the quality of the measurement. Cooling elements such as heatsinks, heatpipes, and cooling ribs are responsible for heat dissipation from the housing. Mechanical elements are necessary for the connection of the principal parts.

The projection unit was built up using a commercially available beamer (HX301G, LG, Seoul, Korea) with a frame rate of 60 Hz. The power consumption of the beamer is less than 100 W and the luminous flux is 300 Lm. The maximal pixel resolution is 1680 × 1050, but for fringe pattern projection the native resolution of 1024 × 768 pixels was used.

**Figure 4:**
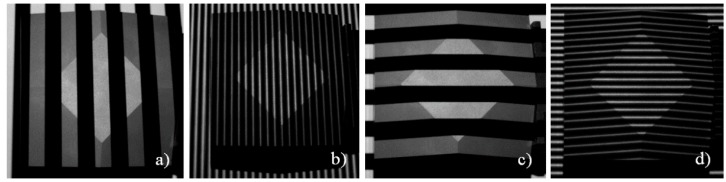
Selected images from fringe sequence: parts of Gray-code sequence (**a**,**c**) and sinusoidal fringes (**b**,**d**) taken from air measurements of a frustum of a pyramid.

A sequence of fringes (Gray-code and sinusoidal fringes) in two orthogonal directions is projected onto the scene by the projection unit (see Figure 4). Typically, a fringe period length of 16, 32, or 64 projector pixels is used. This leads to 64, 32, or 16 vertical and 48, 24, or 12 horizontal projected fringes and seven, six, or five Gray-code images per projection direction. Using a 90° phase shift four sinusoidal fringe images per projection direction are necessary. Hence, one complete projection sequence consists of 24, 22, or 20 single fringe images taking 400, 367, or 333 ms time, respectively.

**Figure 5:**
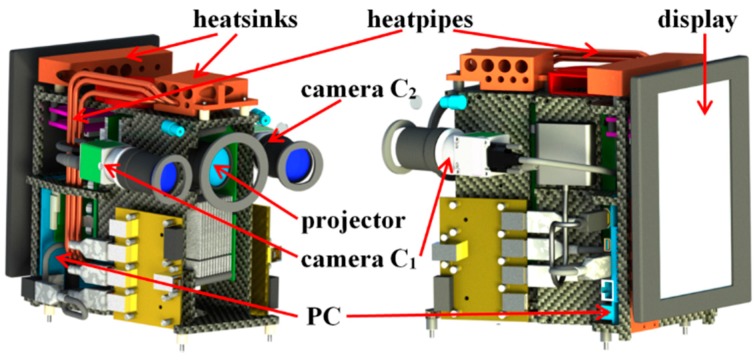
Principal components in two views of a construction drawing.

The fringe image sequence is synchronously recorded by both cameras. From these image sequences the so called phase images are calculated, which are used for the determination of the 3D data (see e.g., [39]). The 3D calculation is performed on the PC, which also has the task to control the image recording. The measurement results are also indicated on the display (see Figure 5). Additional components are two laser pointers for checking the current measurement distance. Figure 5 shows the main components of the scanner in two construction drawings.

The underwater housing was produced using the synthetic material PA 2200 (see [49]) and developed by the 4h Jena Engineering GmbH (see [50]). It can be used in both fresh and salt water. It can distinctly withstand the water pressure at a diving depth of 40 m. The interfaces for the projector, the cameras, and the laser beams were made from sapphire glass, whereas the window for the display is made from polycarbonate. The planar windows for the cameras and the projector do not lie in a common plane but are tilted according to the directions of the optical axis. This should simplify the calibration procedure (see Section 3.5).

**Figure 6:**
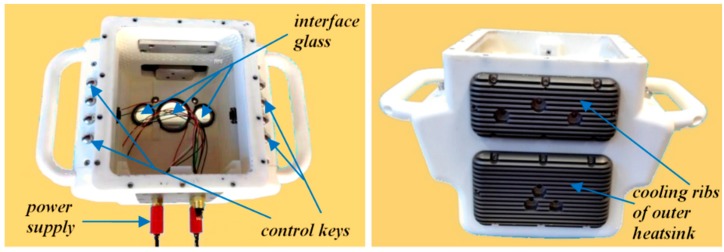
Two housing views showing certain sensor elements.

**Figure 7:**
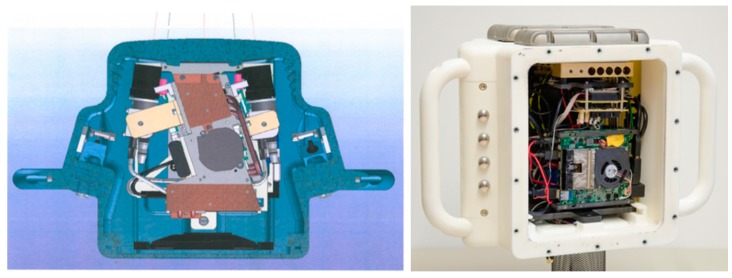
Housing views: construction draw, view from above (**left**), sensor inside housing without back-panel (**right**).

One major problem of the scanner was the adequate heat dissipation from the housing. A base plate with cooling ribs and appropriate heat sinks were constructed. For power supply and signal lines a separable under water plug-in connector cable was selected and employed. Separable inductive switching boards including control keys and interfaces to the scanner were developed in order to provide a correct handling also with diver gloves (see Figure 6). A construction drawing of the scanner is shown in Figure 7 as well as a photograph of the scanner in the housing without back panel with the display.

## 5. Measurements

### 5.1. Calibration Evaluation

**Figure 8:**
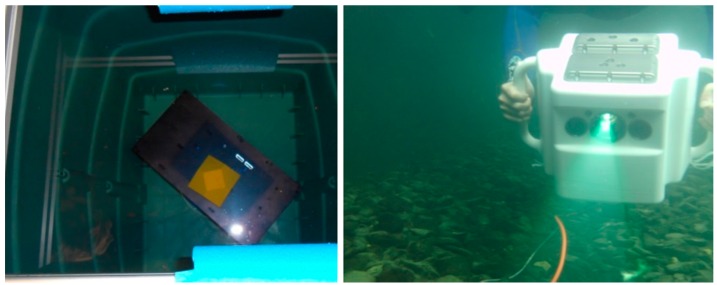
Frustum of pyramid in a rain barrel (**left**) and scanner in underwater use (**right**).

**Figure 9:**
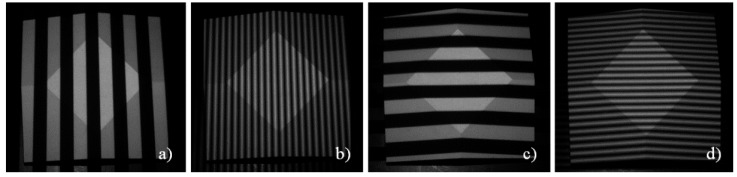
Selected images from fringe sequence: parts of Gray-code sequence (**a**,**c**) and sinusoidal fringes (**b**,**d**) taken from underwater measurements of a frustum of a pyramid.

After the four measurements which were used for the calibration of the underwater parameters we made several underwater measurements of certain specimen in order to evaluate the quality of the calibration. According to the suggestions of the VDI/VDE guidelines [46] the measurement objects ball bar and plane were placed in different positions in the measurement volume. Additionally, a frustum of a pyramid (see Figure 8) was measured. All measurements were performed in clear water using a rain barrel (see Figure 8). Figure 9 shows an example for the projected fringe patterns under water. Compared to Figure 4 differences in the image quality can be hardly detected.

For evaluation the quantities relative length deviation and relative flatness deviation defined by Equations (5) and (6) were used. For comparison, all measurements and calculations were performed

- With the sensor outside the housing in the laboratory (Air)
- Underwater with the pinhole camera model with modified parameters (PM)
- Underwater with the extended model, but without additional distortion matrices (RM)
- Underwater with the extended model including the additional distortion matrices (RMD)

In all cases at least seven measurements were performed. Noise determination was performed additionally. It was defined as standard deviation of the measured 3D points from a small locally fitted plane (or sphere, respectively). The obtained maximum error results are documented by Table 2. Note that for *E_L_* and *E_F_* the percentage values are given in contrast to Equations (6) and (7), and *noise* is an averaged value over all noise measurements.

**Table 2:** Calibration evaluation results (given in %) using specimen ball bar (*E_Lb_*), frustum of pyramid (*E_Lp_*), and plane (*E_F_*), and averaged 3D noise values.

LocationQuantity	*E_Lb_* (%)	*E_Lp_* (%)	*E_F_* (%)	*noise* (mm)
Air, PM	0.1	0.4	0.15	0.02
Water, PM	1.0	1.45	1.7	0.05
Water, RM	0.4	0.6	0.65	0.04
Water, RMD	0.35	0.35	0.4	0.03

The results show that pinhole approximation in water is not sufficient to obtain acceptable measurement accuracy. Although the modeling without additional distortion correction should completely describe the ray geometry, the results are not yet as precise as desired. RMD results are acceptable, but should also be improved at future measurements after certain enhancements which can be obtained, for instance, by using a free ray-based camera model. However, for improved ray-based model representation an additional calibration step must be performed. An approach of this idea will be given in the outlook section.

### 5.2. Examples of Underwater Measurements

**Figure 10:**
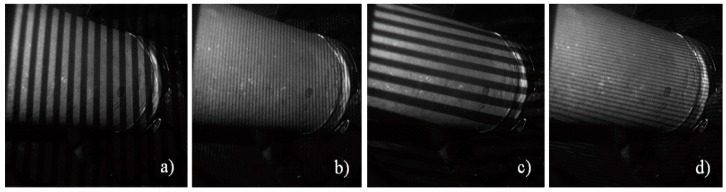
Selected images from fringe sequence: parts of Gray-code sequence (**a**,**c**) and sinusoidal fringes (**b**,**d**) taken from underwater measurements of a pipe.

**Figure 11:**
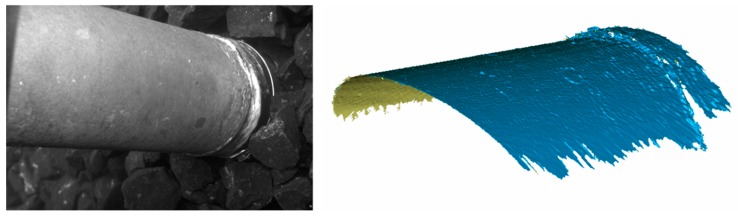
Underwater measurement of a pipe (**left**) and 3D result representation (**right**).

**Figure 12:**
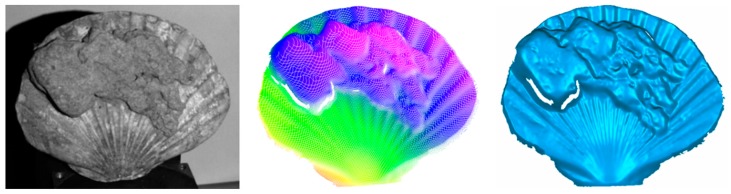
Fossil seashell: photograph (**left**), color-coded 3D- (**middle**), and 3D-surface presentation (**right**).

**Figure 13:**
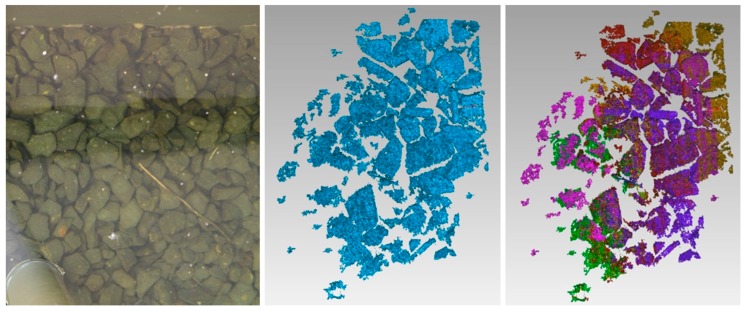
Example underwater measurement stones: photograph (**left**), surface 3D representation (**middle**), and color coding of six different scans (**right**).

Underwater measurements were performed next by application of the scanner in a water basin. It was handled by a diver (Figure 8). The first measurement objects were a pipe (Figure 10 and Figure 11), a fossil sea shell (Figure 12), and stones (Figure 13).

The examples can give a first impression of the capabilities of the underwater scanner. The scan results are obtained few seconds after the recording. It can be assumed that the measurement precision is sufficient for a number of application scenarios. However, for bigger objects the 3D point clouds must be merged. Here, additional software is necessary.

## 6. Summary, Discussion and Outlook

The principle of optical 3D surface measurement based on the fringe projection technique for underwater applications has been described in this work and the challenges of the underwater use of this technique are shown and discussed in contrast to the classical application.

A new fringe projection based 3D underwater scanner which covers a measurement volume of 250 mm × 200 mm × 120 mm for diving depths up to 40 m was introduced. It can record one 3D scan in approximately 350 ms with a lateral resolution of 150 µm in the object space. Measurement objects larger than the measurement volume should be scanned in several views and merged subsequently.

The presented scanning device for 3D surface measurements is one of the first underwater scanners based on fringe projection technique. It is quite complicate to perform a comparison of technical details and measurement capability to other systems based on this technique [18,19,20]. These systems are laboratory setups and the projector is not included in the camera housing. Certainly, what can be assessed is that our scanner has a smaller measurement field. Measurement accuracy, although also difficult to compare, seems to be similar. Compared to photogrammetric underwater systems (e.g., [13,26]) the measurement precision has the same magnitude. The main differences with photogrammetric systems are the smaller measurement volume and the higher measurement point density.

During the image recording time of 0.33 or 0.4 s operator movements may disturb the image recording. Effective movement correction algorithms could manage this, but, unfortunately such methods are not available for this scanner. However, we have developed an algorithm which detects vibrations and movements during the image recording and suggests the rejection of the affected current measurement and its repetition.

If moving dust clouds or dirt particles disturb the image acquisition, it must be decided whether to wait for better recording conditions or to take the images and to accept decreased image quality. In order to make a more exact statement possible, appropriate experiments should be performed in the future.

The first quantitative measurements showed acceptable measurement accuracy. However, best results could be achieved only by application of additional (heuristically found) distortion corrections which cannot be explained by the model. They perhaps compensate partly the effects of the deviations of the current modelling from the real situation. The search of the origins of these effects may be a part of our future work.

First measurements and experiments were performed in clear freshwater in the laboratory and in an outdoor water basin with slightly more pollution. This, however, is not a practice-oriented situation. It is expected that in a sea environment with certain turbidity the robustness of the object surface acquisition will decrease and the noise of the measurement values will increase. Measurement accuracy, however, will be affected only by a slightly higher random error. The corresponding experiments should confirm and quantify these expectations.

The main part of our future work should be occupied by experiments concerning the dependence of the measurement accuracy on the water quality. Here the influence of difference levels of turbidity to the robustness and accuracy of the measurements must be analyzed. Additionally, comparisons between measurements in fresh and salt water will be made.

In order to avoid the necessity of additional distortion compensation by special operator, the complete ray-based representation of the observation system should be used in the future. Here, an additional calibration step with underwater recordings of a plane must be applied similarly to the method described by Bothe *et al.* [37]. With this final transition to the ray-based camera model a further reduction of the remaining length error and flatness deviation should be achieved.

As it was already mentioned before, the size of the measurement volume is not very large and seems to be too small for certain applications such as pipeline system inspection or survey of archaeological sites. The possible stitching of resulting 3D datasets is sophisticated, time consuming, and prone to errors. Hence, scanning devices with considerable larger measurement volume are desired. This requires stronger illumination power of the projection unit with coincident more efficient heat dissipation. The design of such a more powerful underwater 3D scanner will be also a part of our future work. Perhaps, the underwater housing for the projector should be separated from the housing for the cameras because of the heat dissipation.
